# Impacts of Vincristine and Prednisolone Chemotherapy on the Canine Gut Microbiota in Dogs Undergoing Treatment for Lymphoma

**DOI:** 10.1111/vco.13063

**Published:** 2025-05-06

**Authors:** Juan Aragon, Annika M. Weber, Madison Tipton, Jennifer Thomsen, Hend Ibrahim, Kristen Weishaar, Sangeeta Rao, Jan S. Suchodolski, Jonathan Stockman, Elizabeth P. Ryan, Nora Jean Nealon

**Affiliations:** ^1^ Department of Environmental and Radiological Health Sciences College of Veterinary Medicine and Biomedical Sciences; Colorado State University Fort Collins Colorado USA; ^2^ Department of Food Science and Human Nutrition Colorado State University Fort Collins Colorado USA; ^3^ Department of Clinical Sciences College of Veterinary Medicine and Biomedical Sciences; Colorado State University Fort Collins Colorado USA; ^4^ Department of Medical Biochemistry Faculty of Medicine, Zagazig University Zagazig Egypt; ^5^ Department of Clinical Sciences College of Veterinary Medicine and Biomedical Sciences, Flint Animal Cancer Center, Colorado State University Fort Collins Colorado USA; ^6^ Department of Small Animal Clinical Sciences, Gastrointestinal Laboratory College of Veterinary Medicine & Biomedical Sciences, Texas A&M University College Station Texas USA; ^7^ Department of Veterinary Clinical Sciences Lewyt College of Veterinary Medicine, Long Island University Brookville New York USA; ^8^ Department of Veterinary Biomedical Sciences Shreiber School of Veterinary Medicine, Rowan University Glassboro New Jersey USA

**Keywords:** bile acid, canine gut microbiome, chemotherapy, dysbiosis, fatty acid, lymphoma

## Abstract

Chemotherapy can have adverse gastrointestinal effects in dogs and people. The objective of this study was to assess the impact of vincristine and prednisolone/prednisone, as part of a CHOP chemotherapy [cyclophosphamide, hydroxydaunorubicin, oncovin (vincristine) and prednisolone/prednisone] protocol, on gastrointestinal dysbiosis in dogs with lymphoma. We hypothesised the first week of chemotherapy (administration of vincristine and prednisolone/prednisone, VCR/Pred) produces compositional and functional shifts in the canine faecal microbiota that are associated with increased dysbiosis. Faecal samples from canine lymphoma patients (*n* = 25) were compared for microbiota and metabolites before (pre‐chemotherapy) and after the first week of VCR/Pred (post‐chemotherapy). A dysbiosis index (DI) was calculated for each dog via quantitative PCR of seven bacterial taxa established for altered ratios in canine gastrointestinal dysbiosis: *Faecalibacterium, Turicibacter, Escherichia coli, Streptococcus, Blautia*, *Fusobacterium* and *Peptacetobacter hiranonis* (formerly *Clostridium hiranonis* ). There was a significant increase in the DI post‐chemotherapy compared to pre‐chemotherapy (*p* = 0.021) concurrent with a significant decrease in faecal *P. hiranonis* concentrations post‐chemotherapy (*p* = 0.0003). 16S rRNA amplicon sequencing analysis revealed a significant decrease in *Enterococcaceae* post‐chemotherapy (*p* = 0.013). Targeted faecal lipid profiling identified markers of host and bacterial metabolic dysfunction that were altered following chemotherapy, including significant decreases in arachidonate (*p* = 0.0015), nervonate (*p* = 0.027), cholestanol (*p* = 0.011) and campesterol (*p* = 0.0035). These findings support that shifts in gut microbiota structure and function may contribute to gastroenteritis in dogs following the first week of VCR/Pred. Gut dysbiosis measures are important for improved treatment options that alleviate gastrointestinal complications associated with chemotherapy in animals and people.

## 1 Introduction

The lifetime incidence of cancer in dogs is similar to that in people, where one in four dogs will be diagnosed throughout their lifespan [1, 2, 3]. Lymphoma, a cancer of lymphocytes, is one of the most common neoplasias in dogs, with ~1 in every 1000 receiving a diagnosis annually [1, 2, 3]. A mainstay of canine lymphoma treatment is the multiagent CHOP protocol consisting of cyclophosphamide, hydroxydaunomycin (doxorubicin), vincristine and prednisolone/prednisone [4, 5]. Although effective at inducing remission and slowing disease progression, chemotherapy can cause gastrointestinal toxicity that presents as vomiting and diarrhoea [6]. These clinical signs may be distressing to pets and owners, complicate treatment and be a factor in a client's decision to discontinue treatment [7, 8].

The gut microbiota, the microorganisms within the gastrointestinal tract, play critical roles in metabolism and immunity [9]. Consequently, biological (cancer) and/or environmental (chemotherapy) factors can have adverse impacts on gut health and can drive dysbiosis, an imbalance of health‐promoting and disease‐causing microbes with altered microbial function [10, 11]. Dysbiosis has been studied extensively in diseases of animals and people, including colorectal cancer, lymphoma, chronic enteropathies and *Clostridium difficile* diarrhoea [10, 12]. Across diseases, dysbiosis has been associated with acute and chronic diarrhoea [13, 14]. Among canine diarrhoea causes, chemotherapy‐induced gastrointestinal toxicity often leads to dose reductions or treatment interruptions, and it may be a factor in client decision‐making for continued lymphoma treatment [7, 8]. Despite being a routine treatment protocol, little is known regarding the impacts of chemotherapy on the canine gut microbiota structure and function.

The primary objective of this study was to assess how the first week of CHOP treatment (vincristine and prednisolone/prednisone, VCR/Pred) impacts the faecal microbiota of dogs undergoing chemotherapy for lymphoma. An experimental approach integrating a quantitative PCR‐based canine dysbiosis index,16S rRNA sequencing and targeted lipid metabolite profiling was used to examine differences in canine gut microbiota composition and function before and after the first week of VCR/Pred. The central hypothesis is that VCR/Pred chemotherapy causes compositional and functional shifts in the canine faecal microbiota that are associated with increased dysbiosis.

## 2 Materials and Methods

### 2.1 Study Design, Inclusion and Exclusion Criteria

Client‐owned dogs newly diagnosed with lymphoma and scheduled for CHOP chemotherapy (cyclophosphamide, doxorubicin hydrochloride/hydroxydaunorubicin, oncovin (vincristine) and prednisolone/prednisone) were identified through the Colorado State University Veterinary Teaching Hospital Flint Animal Cancer Center (VTH‐ACC) from 2017 to 2019. Inclusion criteria required that all dogs have a new diagnosis of lymphoma made by a licensed veterinarian using a combination of clinical presentation/physical exam findings with cytology, histopathology, flow cytometry and/or polymerase chain reaction for antibody receptor rearrangement. All lymphoma stages were considered for enrollment. Dogs were required to be ≥ 1 year old and have a starting body weight of ≥ 8.0 kg with a body condition score of 4–7 out of 9 on the Nestlé Purina scale [15]. At the time of screening, dogs were not eligible for enrollment (i.e., excluded from enrollment) if they had previously received part or all of a CHOP chemotherapy protocol. Following enrollment, dogs were excluded from downstream analysis if there was a lack of owner compliance (i.e., not providing a pre‐chemotherapy and/or post‐chemotherapy faecal sample). Dogs were not excluded for concurrent disease processes. Diet type, medication and supplement use did not exclude dogs from participation and their use was monitored by a licensed veterinarian throughout participation in this study. Throughout participation in this study, dogs were monitored for the development of adverse health events that were assessed using the Veterinary Cooperative Oncology Group‐Common Terminology Criteria for Adverse Events (VCOG‐CTCAE v2) guidelines [15]. This study was approved by the CSU‐VTH‐ACC Clinical Review Board (VCS #2016‐078).

### 2.2 Canine Lymphoma Chemotherapy Protocol

Chemotherapy schedules were based on protocols described previously [5, 16]. Per the standard protocol, vincristine was administered intravenously at 0.7 mg/m^2^ during weeks 1, 5, 9 and 13. Cyclophosphamide was administered orally at 250 mg/m^2^ during weeks 2, 6, 10 and 14 along with 1–2 mg/kg of oral furosemide. Doxorubicin was given intravenously at 30 mg/m^2^ during weeks 3, 7, 11 and 15. Prednisolone or prednisone was given as a tapering oral course on week 1, starting at ~1 to 2 mg/kg per day. All chemotherapy was prescribed by a licensed veterinarian and administered under the supervision of a registered veterinary technician and/or licensed veterinarian.

### 2.3 Canine Faecal Collection and Processing

At enrollment, a pre‐chemotherapy faecal sample was obtained from each patient. Faecal samples for the post‐chemotherapy timepoint were collected after the first week of the VCR/Pred. Samples were collected as naturally voided faeces and/or during digital rectal examination. Following collection, all samples were individually bagged and stored at −20°C prior to submission for downstream analyses.

### 2.4 Canine Dysbiosis Index Analysis

Canine dysbiosis indices (DI) using quantitative PCR (qPCR) were generated at the Texas A&M University Gastrointestinal Research Laboratory (College Station, TX) as previously described [17]. Data S1 provides additional details for qPCR protocols. DNA extraction from 25 faecal samples (pre‐chemotherapy) and 23 (post‐chemotherapy) was performed using a MoBio powersoil DNA extraction kit following manufacturer protocols. qPCR was performed for the following taxa [18, 19, 20, 21]: Total bacteria, *Faecalibacterium*, *Fusobacteria*, *Blautia*, *Turicibacter*, *Escherichia coli* , *Peptacetobacter* (*Clostridium*) *hiranonis* and *Streptococcus*. DNA concentrations were expressed as log DNA/mg faeces [18, 19, 21]. Concentrations were compared to normal canine reference ranges [17] to make designations of dysbiosis, where increases above the reference range occur with dysbiosis for *E. coli* and *Streptococcus* and decreases occur with dysbiosis for *Faecalibacterium*, *Fusobacteria*, *Blautia*, *Turicibacter* and *P*. *hiranonis*. Total DI values were calculated using centroid algorithms comparing the Euclidian distances of a sample from the centroid of standard healthy versus diseased samples to calculate the DI value [17]. A DI of < 0 was defined as within normal limits (i.e., no dysbiosis), 0–2 indicated mild dysbiosis and > 2 indicated significant dysbiosis [17].

### 2.5 DNA Extraction and Library Preparation for 16S rRNA Amplicon Sequencing

Faeces from 12 dogs, for which there were remaining pre‐ and post‐chemotherapy faeces following DI and metabolomics evaluations, were used for 16S rRNA sequencing analysis. DNA extraction was performed with a MoBio PowerSoil Kit (MoBio Laboratories Inc., Solana Beach, CA) following manufacturer's protocols. Amplification of the V4 region of the 16S rRNA gene was performed using Earth Microbiome Project standard protocols for paired‐end sequencing with the 515F/806R (Parada/Aprill) primer set [22, 23, 24, 25]. Data S1 provides expanded details on 16S rRNA PCR amplification and sequence processing.

### 2.6 16S rRNA Microbiome Analysis and Data Visualisation

Forward and reverse FASTQ files were analysed in R Studio (Version 4.3.2) [26]. Paired‐end sequence reads were trimmed, filtered and converted into amplicon sequence variants (ASVs) using Diverse Amplicon Denoising Algorithm (DADA) 2 [27]. ASVs were taxonomically classified using the SILVA 16S rRNA Sequence Database, version V138.1 [28]. Relative abundances were calculated by normalising ASV read counts to the total number of reads per sample. ASVs representing < 1% of sample relative abundance were filtered from downstream analyses. Alpha and beta diversity were calculated in R using Phyloseq (version 1.40.0) and Vegan (version 2.6–4) [29, 30]. Alpha diversity was measured using Shannon, Inverse Simpson and observed ASV algorithms. Beta diversity was calculated using a Bray–Curtis dissimilarity index and visualised with non‐metric multidimensional scaling (NMDS). The R code that generated this analysis is provided in Data S2.

### 2.7 Targeted GC–MS for Fatty Acids and Bile Acids

To further assess how faecal microbial compositional changes might underscore functional changes in the microbiota in response to chemotherapy, fatty acid, primary and secondary bile acid, and sterol concentrations were established by the Colorado State University Bioanalysis and Omics Facility (Fort Collins, CO) for 46 stool samples, including 23 dogs at the pre‐ and post‐chemotherapy timepoints (RRID SCR_021758). Data S1 provides expanded methods for gas chromatography–mass spectrometry (GC–MS) processing. The following lipids were evaluated: The bile acids cholic acid, chenodeoxycholic acid, lithocholic acid, deoxycholic acid, ursodeoxycholic acid, total primary bile acids, total secondary bile acids, total bile acids; the sterols coprostanol, cholesterol, cholestanol, brassicasterol, lathosterol, campesterol, stigmasterol, fusosterol, beta‐sitosterol, sitostanol and total sterols; the fatty acids myristate, palmitate, linoleate, alpha‐linolenate, oleate, cis‐vaccenate, stearate, arachidonate, gondoate, docosanoate, nervonate and total fatty acids. Metabolite concentrations were expressed as ng (bile acids and sterols) and μg (fatty acids) per mg of faeces.

### 2.8 Statistical Analysis and Data Visualisation

For dysbiosis indices and lipid concentrations, statistical analysis and visualisation were performed using GraphPad Prism (version 10.1.1). A Shapiro–Wilk test was applied to each dataset to assess for normality. Pre and post chemotherapy faecal samples were compared using a paired t test for normally distributed datasets or Wilcoxon matched pairs signed rank test for datasets that were not normally distributed. 16S rRNA data was analysed in GraphPad Prism (version 10.1.1) and R (version 4.3.2). Paired t tests (Shannon, Observed ASVs) and a Wilcoxon matched pairs signed rank test were used to compare alpha diversity metrics in pre‐ versus post‐chemotherapy faecal samples. For beta diversity, permutational analysis of variance (PERMANOVA) was used to compare pre‐ and post‐chemotherapy communities. DESeq2 was used to compare microbial differential abundances at the phylum and family taxonomic levels [31]. A Spearman's correlation was performed between family relative abundances from 16S rRNA amplicon sequencing data and lipid metabolites. Correlation strength was defined using the following criteria: Very weak correlation (*r* = 0.00–0.19), weak correlation (*r* = 0.20–0.49), moderate correlation (*r* = 0.50–0.69), strong correlation (*r* = 0.70–0.89) and very strong correlation (0.90–1.00) [32]. For all tests, significance was defined as *p* < 0.05. All R code is provided in Data S2 (dada2 processing, alpha diversity, beta diversity, differential abundance) and Data S3 (correlation analysis).

## 3 Results

### 3.1 Screening, Enrollment and Patient Demographics

**FIGURE 1:**
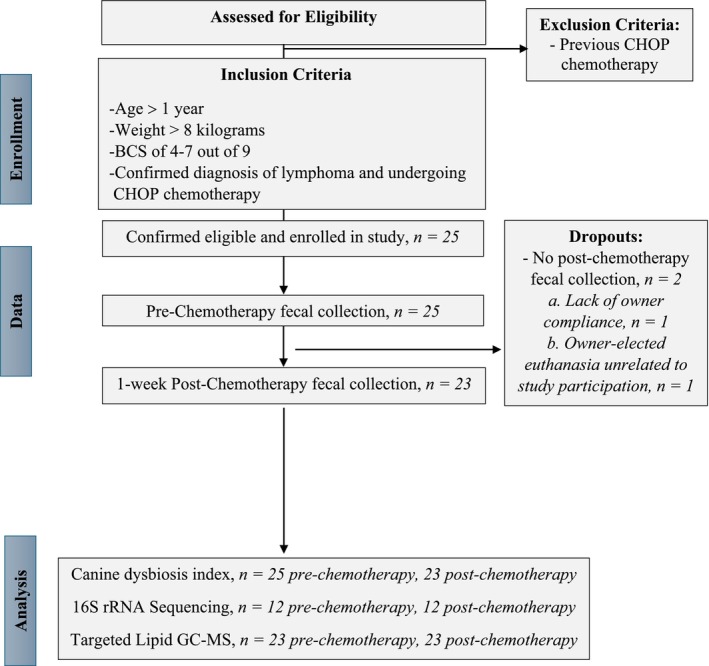
Study design and flow diagram of this prospective, single‐arm (non‐randomised) clinical trial evaluating the faecal microbiota of dogs undergoing chemotherapy for a new diagnosis of lymphoma. CHOP, cyclophosphamide, hydroxydaunorubicin (doxorubicin), oncovin (vincristine) and prednisolone/prednisone; GC–MS, gas chromatography–mass spectrometry.

Figure 1 provides a modified CONSORT flow diagram [33] of this prospective, single‐armed clinical trial and Table 1 provides demographic information for all enrolled dogs and Data S4 provides additional clinical metadata on all enrolled patients, including their age, sex, breed, and information on diet, medication use and comorbidities at the time of enrollment. A total of 25 dogs were successfully enrolled. The patient population consisted of 9 female spayed dogs, 15 male castrated dogs and one intact male dog. Thirteen breeds were represented, including 16 owner‐reported purebred dogs and 9 mixed‐breed dogs. Thirteen dogs were diagnosed with B‐cell lymphoma, and 12 dogs were diagnosed with T‐cell lymphoma. Diet use by patients, when specified, included various commercial dry (extruded kibble) and canned canine diets (*n* = 15), home‐cooked diets (*n* = 2) and raw diets (*n* = 2). Medication, supplement and over‐the‐counter products used by this patient population included amoxicillin and/or amoxicillin clavulanic acid (*n* = 3), anxiolytics and sedatives (*n* = 10), benazepril (*n* = 1), cannabidiol oil supplements (*n* = 5), cough tabs (unspecified formulation) (*n* = 1), dexamethasone sodium phosphate (*n* = 1), joint supplements and polysulfated glycosaminoglycan injections (*n* = 4), maropitant (*n* = 20), metronidazole (*n* = 21), non‐steroidal anti‐inflammatory drugs (*n* = 4), oclacitinib (*n* = 1), omeprazole (*n* = 4), over the counter immune and allergy support supplements (*n* = 3), pimobendan (*n* = 1), sucralfate (*n* = 1), topical, ophthalmic suspensions, otic suspensions and skin wipes (*n* = 3) and thyroxine (*n* = 1).

**TABLE 1:** Summary of patient demographics.

Sex	*n* = 25
Male, castrated	15
Male, intact	1
Female, spayed	9
Breed^a^	*n* = 25
American pit bull terrier	1
Beagle	1
Bernese Mountain Dog	1
Boxer	2
German shepherd	1
Golden retriever	3
Greyhound	1
Labrador retriever	2
Rat terrier	1
Rottweiler	1
Siberian Husky	1
Yorkshire terrier	1
Mixed breed	9
Cancer type	*n* = 25
B‐cell	13
T‐cell	12

^a^ Breed as reported in patient medical record.

Comorbidities at the time of enrollment within this patient population included alopecia (*n* = 2), anaemia (*n* = 6), asymptomatic bacteriuria, symptomatic bacterial urinary tract infection, cystitis and/or urinary incontinence with an open aetiology (*n* = 4), degenerative mitral valve disease (*n* = 2), dental disease (*n* = 5), diarrhoea (*n* = 3), dyspnoea and/or coughing (*n* = 4), electrolyte abnormalities (*n* = 1), elevated liver enzymes (*n* = 8), gingival hyperplasia (*n* = 1), heart murmurs with an open aetiology (*n* = 1), haemangioma, benign, incompletely excised (*n* = 1), hooded vulva (*n* = 2), hypercalcemia (*n* = 3), hygroma (*n* = 1), hyperglobulinemia (*n* = 2), hypoglobulinaemia (*n* = 1), inappetence (*n* = 6), lethargy (*n* = 5), lymphopenia (*n* = 1), mammary gland carcinoma, incompletely excised (*n* = 1), mast cell tumour, incompletely excised (*n* = 1), muscle atrophy (*n* = 2), nasal discharge (*n* = 2), neutrophilia, mature (*n* = 3), ocular disease (*n* = 1), patellar luxation (*n* = 1), renal disease (*n* = 3), seropositive, but asymptomatic, for *Borrelia burgdorferi* antibodies (*n* = 1), orthopaedic diseases (*n* = 5), overweight (BCS 6‐7/9) (*n* = 4), subcutaneous, cutaneous and/or dermal masses with an open aetiology (*n* = 7), polyuria with polydipsia (*n* = 1), proteinuria (*n* = 1), thrombocytopenia (*n* = 4), underweight (BCS 3/9) (*n* = 2), vomiting (*n* = 2) and xeromycteria (*n* = 1) (Data S4).

Two of the 25 enrolled dogs did not complete the study and had a post‐chemotherapy stool sample submitted due to lack of owner compliance (*n* = 1) and owner‐elected euthanasia due to disease progression (*n* = 1). Adverse events reported for patients during participation in this study included anaemia (*n* = 5), dermatitis and/or vasculitis (*n* = 2), diarrhoea (*n* = 11), elevated liver enzymes (*n* = 1), an elevated urine protein to creatinine ratio (*n* = 1), hyperphosphatemia (*n* = 1), hypertension (*n* = 1), inappetence (*n* = 6), increased anxiety (*n* = 1), lethargy (*n* = 3), musculoskeletal pain (*n* = 1), nausea (*n* = 2), pigmenturia (*n* = 1), polycythemia (*n* = 1), polydipsia (*n* = 5), polyphagia (*n* = 2), polyuria (*n* = 5), neutropenia (*n* = 5), urinary incontinence (*n* = 1) and vomiting (*n* = 2) (Data S4). In all instances, adverse events occurred as anticipated adverse events secondary to vincristine, prednisone/prednisolone administration and/or secondary to disease progression from either lymphoma or a comorbid condition, as noted by the licensed veterinarian(s) in the medical record of each patient.

### 3.2 Faecal Dysbiosis Indices Increase Following Vincristine and Prednisolone Chemotherapy in Dogs

**FIGURE 2:**
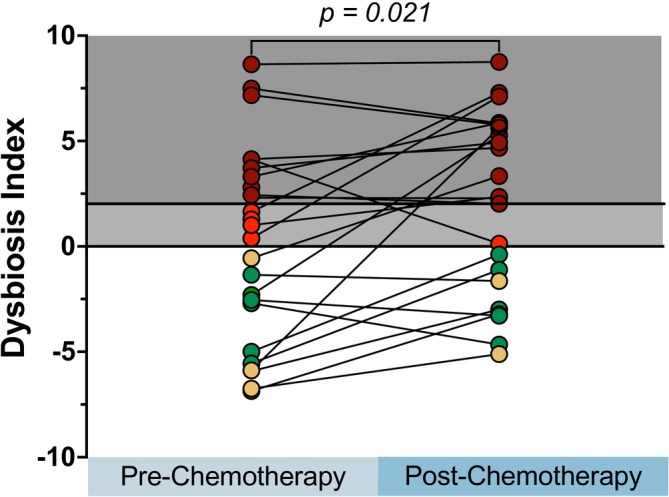
Overall canine microbiota dysbiosis index (DI) values before and after the first week of vincristine and prednisolone/prednisone administration in canine patients (*n* = 25 pre‐chemotherapy samples, *n* = 23 post‐chemotherapy samples). Shaded areas indicate DI standard reference ranges established by the Gastrointestinal Laboratory at the Texas A&M University College of Veterinary Medicine and Biomedical Sciences, where the light‐grey shaded range indicates mild dysbiosis, and the dark‐grey shaded range indicates significant dysbiosis. Circle colours reflect DI classifications, where green indicates “eubiosis”, yellow indicates “subclinical dysbiosis” (overall DI within reference range with one or more indicator taxa outside of reference range), light red indicates “mild dysbiosis” and dark red indicates “significant dysbiosis”. Pre‐ versus post‐chemotherapy dysbiosis indices were compared using a Wilcoxon matched pairs signed rank test, where significance was defined as *p* < 0.05.

Twenty‐five stool samples were collected before chemotherapy (pre‐chemotherapy) and 23 samples were collected following the first week of VCR/Pred (post‐chemotherapy) for canine DI analysis [17]. Figure 2 shows the distribution of dysbiosis indices when comparing faecal samples pre‐ and post‐chemotherapy. When comparing pre‐ and post‐chemotherapy samples, overall DI values significantly increased (*p* = 0.021), shifting from a mean score of 1.01 at the pre‐chemotherapy timepoint to 2.35 at the post‐chemotherapy timepoint. Among pre‐chemotherapy samples, 11 dogs had a DI within normal limits, five had mild dysbiosis and nine had significant dysbiosis. Following the first week of VCR/Pred, eight dogs had a DI within normal limits, one dog had mild dysbiosis and 14 dogs had significant dysbiosis. Among the 11 dogs with a normal DI at the pre‐chemotherapy timepoint, 8 dogs had a normal DI at the post‐chemotherapy timepoint and three dogs shifted from a normal DI to a significantly elevated DI at the post‐chemotherapy timepoint. Among the 14 dogs with significant dysbiosis following the first dose of chemotherapy, three dogs shifted from normal dysbiosis to significant dysbiosis, three dogs shifted from mild dysbiosis to significant dysbiosis and eight dogs had persistently significant dysbiosis at both the pre‐chemotherapy and post‐chemotherapy timepoints.

### 3.3 Vincristine and Prednisolone Chemotherapy Modulates Faecal Abundances of Dysbiosis Index Taxa

**FIGURE 3:**
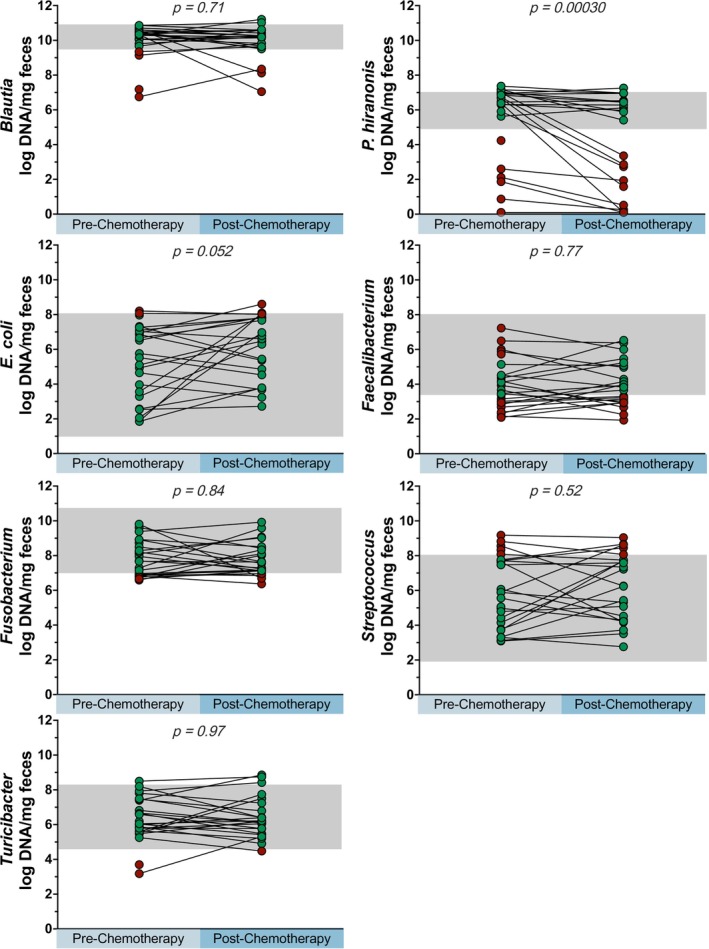
Changes to the log DNA/mg faeces levels of the seven targeted gut microbes evaluated in dysbiosis index (DI) at the pre‐chemotherapy (*n* = 25) and post‐chemotherapy (*n* = 23) timepoints. Shaded areas indicate DI standard reference ranges established by the Gastrointestinal Laboratory at the Texas A&M University College of Veterinary Medicine and Biomedical Sciences. Green circles indicate samples within the established reference range for each taxon and red circles indicate samples outside of the established reference range. *p* values are from paired *t*‐tests (normally distributed samples) and Wilcoxon matched‐pairs signed rank tests (samples not normally distributed), where significance was defined as *p* < 0.05.

The log DNA/mg faeces concentrations of the seven targeted microbial taxa from the DI were further analysed for changes occurring following chemotherapy. Figure 3 shows the log DNA/mg faeces abundance changes of each taxon pre‐ versus post‐chemotherapy. *Peptacetobacter* (formerly *Clostridium*) *hiranonis*, a species that is known to decrease with increasing dysbiosis [17], was significantly lower when comparing post‐chemotherapy to pre‐chemotherapy faecal samples (6.45 log DNA/mg faeces pre‐chemotherapy, 5.41 log DNA/mg faeces post‐chemotherapy, *p* = 0.00030), but was still within the normal reference range (RR = 5.1–7.1 log DNA/mg faeces). *E. coli*, a species that increases with increasing dysbiosis [17], had an increased concentration in post‐chemotherapy samples that trended towards significance (median of 5.76 log DNA concentration pre‐chemotherapy versus 6.60 post‐chemotherapy, *p* = 0.052). No changes in the log DNA/mg faeces abundances of *Blautia* (*p* = 0.71), *Faecalibacterium* (*p* = 0.79), *Turicibacter* (*p* = 0.97), *Fusobacterium* (*p* = 0.84) and *Streptococcus* (*p* = 0.52) were observed when comparing stool samples pre‐ versus post‐chemotherapy.

### 3.4 Vincristine and Prednisolone Chemotherapy Modestly Alters the Canine Faecal Microbiota

**FIGURE 4:**
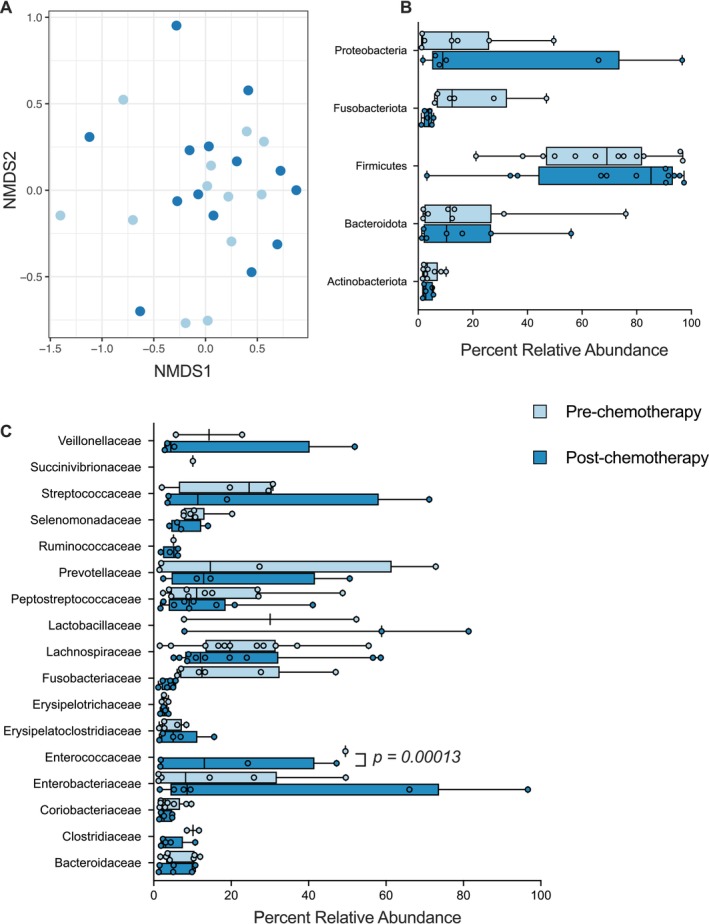
16S rRNA amplicon sequencing analysis of canine cancer patients before (*n* = 12) and after (*n* = 12) 1 week of chemotherapy with vincristine and prednisolone/prednisone. (A) NMDS ordination was calculated using a Bray–Curtis dissimilarity algorithm. Each circle indicates one faecal sample, where dark blue circles indicate pre‐chemotherapy samples and light blue dots indicate post‐chemotherapy samples. Statistical significance for the pre‐versus post‐chemotherapy beta diversity values was calculated using permutational analysis of variance (PERMANOVA), where significance was defined as *p* < 0.05. *R* ^2^ values indicate the percent of sample variation in the model contributed by sample timepoint (i.e., pre‐chemotherapy versus post‐chemotherapy classification). (B) Phylum level changes in the percent relative abundance of bacterial populations before and after 1 week of vincristine and prednisolone/prednisone chemotherapy. (C) Family changes in relative abundance of bacterial populations before and after vincristine and prednisolone/prednisone chemotherapy. Box plots represent median and range, where individual circles in each bar indicate individual samples. Abbreviations: NMDS, non‐metric multidimensional scaling.

16S rRNA analysis was performed on a subset of dogs for which faeces were readily available for both the pre‐chemotherapy (*n* = 12) and post‐chemotherapy (*n* = 12) samples (Figure 4). When comparing the pre‐ versus post‐chemotherapy faecal microbiota communities, there were no significant differences in alpha diversity (Shannon, *p* = 0.16; Inverse Simpson, *p* = 0.11; and observed ASVs, *p* = 0.43), or in beta diversity (*p* = 0.065) (Figure 4A). Changes in microbial differential abundance were further assessed at the phylum and family taxonomic levels (Figure 4B,C, Data S5). No phyla were differentially abundant when comparing pre‐ versus post‐chemotherapy samples. At the family level, chemotherapy was associated with a significant decrease in *Enterococcaceae* (1.38‐fold decrease post‐chemotherapy versus pre‐chemotherapy, *p* = 0.00013).

### 3.5 Vincristine and Prednisolone Chemotherapy Drives Changes in Faecal Bile Acid, Sterol and Fatty Acid Concentrations

Given that both microbiome composition and function can explain health and disease states [10, 11, 34], stool samples were analysed with targeted GC–MS to compare concentrations of bile acids, sterols and fatty acids pre‐ versus post‐chemotherapy. A total of 46 stool samples were used, including 23 pre‐chemotherapy and 23 post‐chemotherapy samples. Table 2 provides median values, ranges and *p* values for pre‐ versus post‐chemotherapy metabolite concentrations. Three lipid metabolites were differentially abundant when comparing pre‐ versus post‐chemotherapy timepoints. These included decreases in the fatty acids arachidonate (median of 1.32 μg/mg faeces pre‐chemotherapy, 1.05 μg/mg post‐chemotherapy, *p* = 0.0015), as well as nervonate (0.60 μg/mg faeces pre‐chemotherapy, 0.55 μg/mg faeces post‐chemotherapy, *p* = 0.027). Concentrations of cholestanol, a sterol, were significantly decreased post‐chemotherapy (0.13 μg/mg pre‐chemotherapy, 0.09 μ/mg post‐chemotherapy, *p* = 0.011). There were no statistically significant changes in primary or secondary bile acids.

**TABLE 2:** Faecal lipid concentrations pre‐ versus post‐chemotherapy.

Chemical class	Metabolite	Pre‐chemotherapy (ng/mg)	Post‐chemotherapy (ng/mg)	*p*
Primary bile acid	Chenodeoxycholic acid	262.10 (2.52–1797.00)	293.90 (4.06–2335.00)	0.84
	Cholic acid	1430.00 (8.33–16922.00)	2178 (15.30–14235.00)	0.70

Chemical class	Metabolite	Pre‐chemotherapy	Post‐chemotherapy	*p*
Secondary bile acid	Deoxycholic acid	2586.00 (252.9–11165.00)	1652.00 (230.40–13312.00)	0.25
	Lithocholic acid	499.00 (33.61–7698.00)	102.10 (32.82–5154.00)	0.15
	Ursodeoxycholic acid	70.25 (26.89–282.30)	42.14 (25.65–369.70)	0.75

	Metabolite	Pre‐chemotherapy (g/mg)	Post‐chemotherapy (g/mg)	*p*
Fatty acid	α‐Linoleate (18:3 n‐3)	1.23 (0.23–5.54)	1.13 (0.29–10.97)	0.84
	Arachidonate (20:4 n‐6)	**1.32 (0.67–2.14)**	**1.05 (0.53–2.97)**	**0.0015**
	Cis‐vaccenate (18:1 n‐7)	0.74 (0.036–14.96)	1.01 (0.10–4.52)	0.18
	Docosanoate (22:0)	0.46 (0.30–0.99)	0.43 (0.22–1.03)	0.052
	Erucate (22:1 n‐9)	0.18 (0.086–0.44)	0.15 (0.022–0.98)	0.25
	Gondoate (20:1 n‐9)	0.31 (0.085–2.93)	0.26 (0.16–0.97)	0.50
	Linoleate (18:2 n‐6)	7.49 (1.85–19.25)	6.43 (2.15–25.23)	0.96
	Myristate (14:0)	0.50 (0.14–1.50)	0.51 (0.18–2.19)	0.77
	Nervonate (24:1 n‐9)	**0.60 (0.38–1.56)**	**0.55 (0.37–1.17)**	**0.027**
	Palmitate (16:0)	6.27 (3.87–26.83)	7.20 (2.44–15.20)	0.77
	Oleate (18:1 n‐9)	6.64 (0.37–24.83)	8.11 (0.30–18.93)	0.52
	Stearate (18:0)	3.37 (1.50–22.82)	3.23 (1.51–16.75)	> 0.99

Chemical class	Metabolite	Pre‐chemotherapy (g/mg)	Post‐chemotherapy	*p*
Phytosterol	Beta‐sitosterol	0.86 (0.42–1.64)	0.91 (0.60–1.09)	0.30
	Brassicasterol	0.049 (0.011–0.19)	0.043 (0.0028–0.18)	0.41
	Campesterol	**0.63 (0.25–0.68)**	**0.52 (0.33–0.75)**	**0.035**
	Cholestanol	**0.13 (0.029–0.47)**	**0.099 (0.034–0.29)**	**0.011**
	Coprostanol	0.021 (0.00–1.73)	0.043 (0.0041–0.37)	0.21
	Fusosterol	0.077 (0.021–0.21)	0.084 (0.038–0.21)	0.62
	Lathosterol	0.035 (0.0052–0.22)	0.016 (0.0038–0.19)	0.11
	Sitostanol	0.069 (0.022–0.31)	0.077 (0.029–0.29)	0.82
	Stigmasterol	0.31 (0.11–0.64)	0.27 (0.17–0.45)	0.31

*Note*: Comparison of pre‐chemotherapy (*n* = 23) versus post‐chemotherapy (*n* = 23) concentrations of selected faecal fatty acids, bile acids and sterols. Pre‐ versus post‐chemotherapy concentrations were compared using either a paired *t*‐test (normally distributed samples) or pairwise Wilcoxon test (samples did not meet normality assumptions). Metabolite concentrations are shown as a median (range). Bolded metabolites were statistically significant, where significance was defined as *p* < 0.05.

Abbreviations: g, gram; mg, milligram; ng, nanogram.

### 3.6 Correlation of Dysbiosis Index Values With Lipid Metabolites

**FIGURE 5:**
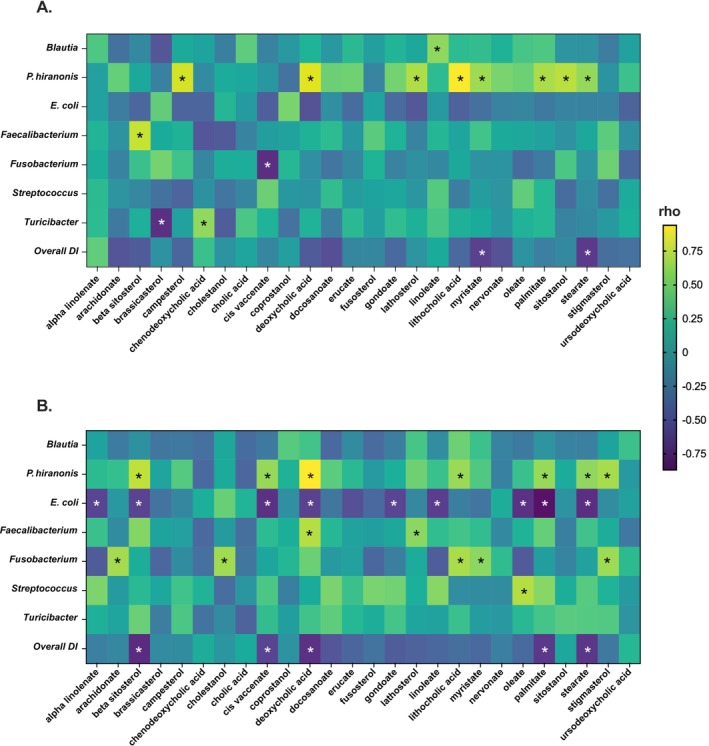
Spearman correlation matrix heatmap evaluating the associations between dysbiosis index (DI) parameters and lipid metabolites that were statistically significant in (A) pre‐chemotherapy (*n* = 12) and (B) post‐chemotherapy (*n* = 12) timepoints. Each cell represents the rho value for the specified metabolite × DI marker pair. Box colours indicate correlation strength and direction. Asterisks (*) indicate statistically significant correlations, where significance was defined as *p* < 0.05 following Spearman's correlation analysis testing.

To understand the relationships between microbial abundances and metabolite concentrations, Spearman correlations were performed between lipid concentrations and the log DNA/mg faeces values of all DI panel taxa (Figure 5, Data S6). Among pre‐chemotherapy samples, 13 lipids were significantly correlated with one or more DI taxa, including three lipids strongly to very strongly correlated with *P. hiranonis*. For *P. hiranonis*, this included a very strong positive correlation with the secondary bile acid lithocholic acid (*r* = 0.92, *p* = 1.19 E‐5) and strong positive correlations with the secondary bile acid deoxycholic acid (*r* = 0.83, *p* = 0.00065) and the sterols campesterol (*r* = 0.79, *p* = 0.0018) and sitostanol (*r* = 0.71, *p* = 0.0082). Additional correlations at the pre‐chemotherapy timepoint included a strong positive correlation between *Faecalibacterium* and the sterol beta‐sitosterol (*r* = 0.75, *p* = 0.0044) and strong negative correlations between *Turicibacter* and the sterol brassicasterol (*r* = −0.72, *p* = 0.0072) and between *Fusobacterium* and the fatty acid cis‐vaccenate (*r* = −0.74, *p* = 0.057). No lipids at the pre‐chemotherapy timepoint significantly correlated with *E. coli* or *Streptococcus*.

For post‐chemotherapy samples, 15 lipids were significantly correlated with one or more DI taxa. Two lipids were very strongly to strongly correlated with *P. hiranonis*, and four lipids strongly correlated with *E. coli* . For *P. hiranonis*, this included a very strong positive correlation with the secondary bile acid deoxycholic acid (*r* = 0.93, *p* = 6.25 E‐6) and a strong positive correlation with the sterol beta‐sitosterol (*r* = 0.73, *p* = 0.0061). *For E. coli*, there were strong negative correlations with the fatty acid palmitate (*r* = −0.86, *p* = 0.0026), stearate (*r* = −0.73, *p* = 0.0066), oleate (*r* = −0.71, *p* = 0.0083) and cis‐vaccenate (*r* = −0.72, *p* = 0.0082). Additional correlations included strong negative correlations between overall DI values with beta‐sitosterol (*r* = −0.74, *p* = 0.0052) and for overall DI values with deoxycholic acid (*r* = −0.71, *p* = 0.0085) and strong positive correlations between *Faecalibacterium* and deoxycholic acid (*r* = 0.70, *p* = 0.010) and between *Streptococcus* and oleate (*r* = 0.70, *p* = 0.010).

## 4 Discussion

The objective of this study was to evaluate the impacts of the first week of CHOP chemotherapy (vincristine and prednisolone/prednisone) on the faecal microbiota and lipid metabolites of client‐owned dogs newly diagnosed with lymphoma [3]. While chemotherapy is successful at inducing remission for many patients, owners often report adverse gastrointestinal effects in their pets [8]. Furthermore, it is currently unknown how chemotherapy impacts the gut microbiota both acutely and long‐term. Evaluating changes in the gut microbiota that occur at the start of chemotherapy treatment is consequently important to consider, given the growing role of the gut microbiota in disease prevention, progression and treatment response.

The current study applies a previously established DI, validated for use in dogs with chronic enteropathies [17], to analyse gut microbiota changes in canine lymphoma patients and evaluate it alongside 16S microbiome sequencing of stool samples. A significant increase in the overall DI was observed when comparing pre‐chemotherapy to post‐chemotherapy samples, supporting that there is an adverse shift within the gut microbiome following the first week of VCR/Pred treatment. An increased overall DI value has been previously seen in dogs with multicentric B‐cell lymphoma when compared to healthy dogs [12], indicating that lymphoma itself may contribute to adverse shifts in the microbiota. Similarly, 14 of the 25 dogs in this study had a DI mildly to markedly increased before starting chemotherapy (Figure 2). Given that, for all dogs, the overall DI either increased in severity or remained within the same state following the first week of chemotherapy, it can be suggested that the synergistic impacts of both lymphoma and chemotherapy are driving dysbiosis in dogs receiving VCR/Pred for lymphoma treatment.

Alongside an increase in overall dysbiosis at the post‐chemotherapy timepoint, there were significant shifts to individual taxa on the DI panel (Figure 3). Post‐chemotherapy, *P. hiranonis* concentrations significantly decreased and *E. coli* concentrations trended towards a significant increase. *P. hiranonis* is a key gut microbiota species responsible for the conversion of primary bile acids into secondary bile acids [35]. A decrease in *P. hiranonis* results in decreased conversion of primary bile acids to secondary bile acids, which can result in the proliferation of enteropathogens and resultant dysbiosis [36]. *E. coli* normally persists as a gut commensal [37]. However, intestinal *E. coli* populations can pathogenically expand due to environmental changes, including insults such as lymphoma and chemotherapy [38]. Given the increases in *E. coli* and decreases in *P. hiranonis* noticed at the post‐chemotherapy timepoint, it may be understood that these two microbiota populations are driving the increase in DI values recorded at the post‐chemotherapy timepoint.

Global 16S rRNA sequencing of the gut microbiota pre‐ and post‐chemotherapy (Figure 4) showed no changes to alpha diversity or beta diversity, suggesting that VCR/Pred does not appreciably alter the overall gut microbial community. Differential abundance testing performed at the phylum and family taxonomic levels provided additional support for stability in the overall microbiota structure, yet the family *Enterococcaceae* did significantly decrease post‐chemotherapy when compared to pre‐chemotherapy (Data S5). This finding has translational significance as previous studies demonstrated that children undergoing chemotherapy for acute lymphoblastic leukaemia had increased levels of *Enterococcaceae* that were associated with an increased risk of subsequent opportunistic infections [39]. While the clinical significance of this decrease in dogs is unclear, evaluating faecal *Enterococcaceae* levels throughout chemotherapy may have utility in anticipating adverse effects of chemotherapy and allow for earlier intervention and treatment.

Alongside potential changes to gut microbial composition, dysbiosis drives changes in microbial function [10]. Given the roles of the gut microbiota in the metabolism of fatty acids, sterols and bile acids [10, 34], functional analysis herein focused on targeted metabolomics panels spanning these metabolites. Among fatty acids, faecal arachidonate concentrations significantly decreased post‐chemotherapy and were significantly correlated with concentrations of *Faecalibacterium* and *Fusobacterium* (Table 2, Figure 5, Data S6). Arachidonate is a host‐derived precursor in multiple pro‐inflammatory cascades, and arachidonic acid metabolising enzymes and their products have been associated with cancer initiation, promotion and progression pathogeneses [40]. Previously, the probiotic *Faecalibacterium prausnitzii* was shown to increase arachidonate production in vivo [41], supporting that there is a gut microbial component to arachidonate metabolism. The reduction in this metabolite after the first week of VCR/Pred chemotherapy indicates a potential state of metabolic dysbiosis that could be targeted in future therapeutics, and it illustrates the complex interactions between the gut microbiota and host metabolism driving inflammation.

Among bile acids and sterols, campesterol and cholestanol decreased post‐chemotherapy. Campesterol is a plant phytosterol that regulates bile acid metabolism via modulation of cholesterol levels, where cholesterol is an upstream precursor in primary bile acid synthesis [42, 43]. Cholestanol is a host and microbial‐derived sterol that is an intermediate in cholesterol metabolism modulating the flux of cholesterol into bile acid synthetic pathways [44, 45]. It is possible that decreases in sterol concentrations, which are indirectly reflective of changes in bile acid metabolism, are related to concentrations of *P. hiranonis*, which was significantly positively correlated with campesterol pre‐chemotherapy (Figure 5, Data S6). In dogs, *P. hiranonis* is instrumental in the conversion of primary bile acids to secondary bile acids and is decreased in dogs with intestinal dysbiosis [46], suggesting depletion of *P. hiranonis* and/or cholestanol may be key contributors to the dysbiosis observed in several dogs herein.

This study had several limitations. The faecal samples analysed were collected only after completion of the first week of CHOP chemotherapy, where VCR/Pred was administered. Therefore, this analysis does not examine how cyclophosphamide, doxorubicin and repeated exposures to prednisolone/prednisone and vincristine impact the gut microbiota, nor can this analysis discern the individual contributions of prednisolone/prednisone versus vincristine on the microbiome, since they were given concurrently to all patients in this study. The variety of diets used among patients may additionally have impacted pre‐ versus post‐chemotherapy microbiota and metabolite evaluations. Diet‐driven changes in gut microbiota composition and function are widely reported in dogs and people [47, 48]. Additionally, medication use by patients at the time of enrollment and/or in the weeks prior to enrollment, including glucocorticoids and antimicrobial drugs, which are known to alter the canine gut microbiota, could have contributed to additional impacts on the pre‐chemotherapy faecal microbiota [49, 50]. Consequently, it is difficult to determine the full impact of factors causing variation in dysbiosis changes for dogs in this study. DI analysis revealed that multiple dogs were in states of significant dysbiosis prior to initiation of chemotherapy (Figure 2). This could indicate that underlying subclinical diseases not accounted for, along with lymphoma, were simultaneously impacting the gut microbiota before, during and after chemotherapy. It is currently unclear whether these concurrent diseases contribute to similar versus different dysbiosis states in the canine gut microbiota. Additionally, there was variation in how the dysbiosis state changed after the first week of chemotherapy. In some dogs, dysbiosis increased to a state of significant dysbiosis; in others, it increased to a state of moderate dysbiosis, and some dogs remained in a state of normal dysbiosis, indicating differential responses of the gut microbiota to chemotherapy. Furthermore, differences in microbial composition were seen pre‐ versus post‐chemotherapy with the DI, but the 16S microbiota was largely unchanged. While the DI is a targeted, quantitative approach for assessing the microbiota, taxonomic identification via 16S sequencing is significantly impacted by ASV processing methods, influenced by the taxonomic classification library, and has limited resolution at higher taxonomic levels, including genus and species, which were profiled on the DI [17, 51]. Given these diverse outcomes, it is therefore important to recognise that personalised medicine approaches incorporating multiple, parallel analyses of gut microbial composition and function may be needed to best support each patient's unique microbiota and its responses to chemotherapy.

This study demonstrates that chemotherapy with VCR/Pred mediated exacerbation of gut dysbiosis and impacts microbial function in dogs with lymphoma. The microbiota and lipid metabolic shifts are identified as contributing factors. Further studies validating the DI for use in canine lymphoma patients, including as a tool to monitor gut dysbiosis during chemotherapy, can be explored to guide treatment decisions. The results from this study illustrate the need to incorporate gut microbiota‐modulatory treatments into chemotherapy protocols, including diet interventions, probiotics, prebiotics and/or faecal microbiota transplantation. The results from this study additionally highlight the need to examine the impact of individual chemotherapy agents on the canine gut microbiota and any additive or synergistic effects they may have as parts of multimodal chemotherapy regimens. Supporting gut microbiota during chemotherapy may be important for improving quality of life, willingness to continue care and can be used to guide treatment modifications that benefit long‐term gut health.

## Conflicts of Interest

Dr. Jan Suchodolski is employed by the Gastrointestinal Laboratory at Texas A&M University, which provides gastrointestinal function assays, including the Dysbiosis Index, on a fee‐for‐service basis. Dr. Jonathan Stockman is a paid consultant for Petco Health and Wellness Company and has previously received a speaking honorarium from Hill's Pet Nutrition and research support from Hill's Pet Nutrition and Royal Canin, a subsidiary of Mars. The remaining authors declare no conflicts of interest. None of the above declarations, for either author, impacted the content of this manuscript.

## Supporting information


**Data S1.** Expanded methods for dysbiosis index, 16S rRNA amplicon sequencing and targeted lipid GC–MS sample processing and analysis.

**Data S2.** R code used to process 16S rRNA amplicon sequencing data.

**Data S3.** R code used to process Spearman’s correlation analysis.

**Data S4.** Patient demographic and clinical data summarised for individual patients.

**Data S5.** 16S rRNA relative abundance and differential abundance values and summary statistics for all phylum and family taxa at the pre‐chemotherapy and post‐chemotherapy timepoints.

**Data S6.** Rho (*r*‐values) and *p* values for Spearman’s correlations between DI taxa and metabolite concentrations at the pre‐chemotherapy and post‐chemotherapy timepoints.

## Data Availability

Raw sequences and selected metadata for the 16S rRNA faecal microbiota samples that support the findings of this study are openly available through the National Center for Biotechnology Information Sequence Read Archive (NCBI SRA via the BioProject number PRJNA976481).
