# Supplementary material for: Impacts of Vincristine and Prednisolone Chemotherapy on the Canine Gut Microbiota in Dogs Undergoing Treatment for Lymphoma
Source: Vet Comp Oncol. 2025 May 6;23(3):388–400. doi: 10.1111/vco.13063 (PMC12353578; doi:10.1111/vco.13063)
Supplement: Supplementary file 1 — Data S1. Expanded methods for dysbiosis index, 16S rRNA amplicon sequencing and targeted lipid GC–MS sample processing and analysis. [file VCO-23-388-s006.docx]

**File S1.** Expanded Methods for Dysbiosis Index, 16S rRNA Amplicon Sequencing Analysis, and Targeted GC-MS Lipid Analysis

**Canine Dysbiosis Index Analysis**

Canine dysbiosis indices (DI), which use quantitative PCR (qPCR) to evaluate the balance of health and disease-causing microbial taxa, were generated for fecal samples at the Texas A&M University Gastrointestinal Research Laboratory (College Station, TX, USA) using previously published methods^16^. Samples from 25 dogs (pre-chemotherapy) and 23 dogs (post-chemotherapy) were sent on dry ice and stored at -80°C until analysis. DNA extraction was performed using a MoBio powersoil DNA extraction kit following manufacturer protocols. qPCR was performed for the following microbial taxa ^17–20^: Total bacteria, *Faecalibacterium*, *Fusobacteria*, *Blautia*, *Turicibacter*, *Escherichia coli*, *Peptacetobacter* (*Clostridium*) *hiranonis*, and *Streptococcus*. qPCR reactions consisted of the following components: 2 µL of 5 ng/µL extracted sample DNA, 5 µL SoFast EvaGreen supermix (Bio-Rad Laboratories Inc., Des Plains, IL, USA), 0.4 µL each of forward and reverse primer prepared to a final concentration of 400 nM., and 2.6 µL of PCR-grade water. Primers consisted of the following sequences (written 5’ to 3’): Total bacteria CCTACGGGAGGCAGCAGT (forward), ATTACCGCGGCTGCTGG (reverse); *Faecalibacterium* GAAGGCGGCCTACTGGGCAC (forward), GTGCAGGCGAGTTGCAGCCT (reverse); *Fusobacteria* KGGGCTCAACMCMGTATTGCGT (forward), TCGCGTTAGCTTGGGCGCTG  (reverse); *Blautia* TCTGATGTGAAAGGCTGGGGCTTA (forward), GGCTTAGCCACCCGACACCTA (reverse); *Turicibacter* CAGACGGGGACAACGATTGGA (forward), TACGCATCGTCGCCTTGGTA (reverse); *E. coli* GTTAATACCTTTGCTCATTGA (forward), ACCAGGGTATCTAATCCTGTT (reverse); *P. hiranonis* AGTAAGCTCCTGATACTGTCT (forward), AGGGAAAGAGGAGATTAGTCC (reverse); Streptococcus TTATTTGAAAGGGGCAATTGCT (forward), GTGAACTTTCCACTCTCACAC
(reverse).
 Cycle conditions for qPCR consisted of the following steps: 120 seconds of initial denaturation at 98°C followed by 40 consecutive cycles of denaturation at 98°C for 3 seconds, annealing for 3 seconds (temperature range of 50°C-63°C varying for each specific taxa primers)^16^. Following amplification, melt curve analysis was performed for each sample using the following steps: 95°C for 60 seconds followed by 55°C for 60 seconds, and then followed by 0.5°C incremental increases every 5 seconds for 80 cycles. Concentrations were expressed as log DNA for each taxa / 10 ng extracted fecal DNA^17,18,20^. These concentrations were compared to normal canine reference ranges ^16^ to make designations of dysbiosis, where increases above the reference range are seen with dysbiosis for *E. coli* and *Streptococcus* and decreases below the reference range are seen with dysbiosis for *Faecalibacterium*, *Fusobacteria*, *Blautia*, *Turicibacter*, and *P. hiranonis*. Total dysbiosis index values were calculated using centroid algorithms that compare the Euclidian distances of a sample from the centroid of standard healthy versus diseased samples to calculate the DI value^16^. A DI of < 0 was defined as within normal limits (i.e. no dysbiosis), a DI of 0-2 indicated mild dysbiosis, and a DI of >2 indicated significant dysbiosis^16^.

**DNA extraction and library preparation for 16S rRNA amplicon sequencing**
 Feces from 13 dogs, for which there was remaining pre- and post-chemotherapy feces following dysbiosis index and metabolomics evaluations, were additionally used to conduct 16S rRNA amplicon sequencing analysis. DNA extraction using a MoBio PowerSoil Kit (MoBio Laboratories Inc., Solana Beach, CA, USA) following manufacturer protocols. Amplification of the V4 region of the 16S rRNA gene was performed using Earth Microbiome Project standard protocols for paired end sequencing with the 515F/806R (Parada/Aprill) primer set^21–24^. The sequence for the forward primer (written 5’ to 3’) was: GTGYCAGCMGCCGCGGTAA^21^. The sequence for the reverse primer (written 5’ to 3’) was: GGACTACNVGGGTWTCTAAT^22^ PCR conditions included the following steps: a one-time, 15-minute denaturation step at 95°C, followed by 34 cycles of 30 seconds of denaturation at 94°C with 90 seconds of primer annealing at 92°C and 60 seconds of DNA polymerization at 72°C. Following these cycles, all amplicons underwent extension for 10 minutes at 72°C. Quality checking of amplicons was performed using gel electrophoresis on 1.5% agarose gel (BioRad, Hercules, CA) with 1 µL Ethidium Bromide added to samples for visualization (Thermo-Fisher Scientific, Lafayette, CO). PCR-grade water was used as a negative control to confirm sample purity. DNA quantification was performed using a NanoDrop 2000 (Thermo-Fisher Scientific). A pooled library of amplicon sequences was created using 50 ng of DNA from each sample. Sequencing was performed on an Illumina Miseq apparatus (Illumina Inc., San Diego, CA) using V2 chemistry with 500 cycles (2x 250 paired end approach). A 15% PhiX mock library was included in the sequencing run to minimize read clustering errors.

**16S rRNA microbiome analysis and data visualization**
 Forward and reverse FASTQ files were imported and analyzed in R Studio (Version 4.3.2)^25^. Paired-end sequence reads trimmed, filtered, and converted into amplicon sequence variants (ASVs) using Diverse Amplicon Denoising Algorithm (DADA) 2^26^. Prior to taxonomic classification, chimera sequences were removed, along with ASVs < 250 base pains or > 256 base pairs in length. The ASVs were taxonomically classified using the SILVA 16S rRNA Sequence Database, version V138.1^27^. ASV relative abundances were calculated by normalizing ASV read counts to the total number of read counts in each sample. ASVs representing <1% of sample relative abundance were filtered from all proceeding downstream analyses. Alpha diversity and beta diversity were calculated in R using the Phyloseq (version 1.40.0) and Vegan (version 2.6-4) packages^28,29^. Alpha diversity was measured using Shannon, Inverse Simpson, and observed ASV algorithms. Beta diversity was calculated using a Bray-Curtis dissimilarity index and visualized with non-metric multidimensional scaling (NMDS). The R code used to generate this analysis is provided in **Supplementary File 2**.

**Targeted GC-MS for fatty acids and bile acids**
 Fatty acid, primary bile acid, and secondary bile acid concentrations were established by the Colorado State University Bioanalysis and Omics Facility (Fort Collins, CO, USA) for 46 stool samples, 23 dogs at both the pre- and post-chemotherapy timepoints (RRID SCR_021758). Lipids were extracted and derivatized to their methyl esters for analysis by gas chromatography coupled with mass spectrometry (GC-MS). For each sample, 80 mg of lyophilized stool was mixed with 1.2 mL of methyl tert-butyl ether/methanol (2:1 v/v) for 1h at 4°C, and then centrifuged at 2,000 g x 15 min at 4°C. Approximately 1 mL of each sample supernatant was recovered, and the solvent was removed under nitrogen gas. The dried extracts were added with 100 µL toluene and 200 µL of 3 N methanolic hydrochloric acid (Sigma-Aldrich) and incubated at 60°C for 2 h. The resulting fatty acid methyl esters were extracted with 700 µL of water and 300 µL of hexane. The hexane phase was recovered, dried under nitrogen, and resuspended in 1 mL of hexane for stool samples. Quality control samples were pooled from the hexane extract of each sample. Hexane extract (1 µL) was injected into a DB-WAX-UI column (30 m x 0.25 mm x 0.25 µm, Agilent) in a Trace1310 GC (Thermo) coupled to a Thermo ISQ-LT MS. The injector temperature was 250˚C, and split ratio was 25:1. A constant flow rate of the carrier gas (He) was controlled at 1.2 mL/min. The initial oven temperature was 150˚C and held for 0.5 min, then increased to 250˚C at 10˚C/min and held for 6 min. Detection was completed under electron impact mode, with a scan range of 50-550 atomic mass units and a scan rate of 5 scans/second. Transfer line and source temperature were both at 250˚C. Data processing was completed with Chromeleon 7 software (Thermo). To control for spectral drift and batch effects, quality control samples were injected after every 6 samples.

Each target analyte was visually inspected for retention time and peak area integration. Peak areas from total ion chromatograph were included for major fatty acids that are high in abundance with good peak shape and were not interfered by background noise or overlapping signals. Peak areas of minor fatty acids were taken as intensities of extracted ions. The retention time of most fatty acids were confirmed by comparing with commercial standards (GLC-85, NuChek). Fatty acids absent in the standards were determined by comparing their spectra to in silico spectral libraries while accounting for their retention time relative to other confirmed fatty acids. Lipid concentrations were inferred via comparison to standard calibration curves.

The following lipids were evaluated on the GC-MS panel: The bile acids cholic acid, chenodeoxycholic acid, lithocholic acid, deoxycholic acid, ursodeoxycholic acid, total primary bile acids, total secondary bile acids, total bile acids, the sterols coprostanol, cholesterol, cholestanol, brassicasterol, lathosterol, campesterol, stigmasterol, fusosterol, beta-sitosterol, sitostanol, and total sterols, the fatty acids myristate, palmitate (16:0), linoleate (18:2 n-6), alpha-linolenate (18:3 n-3), oleate (18:1 n-9), cis-vaccenate (18:1, n-7), stearate (18:0), arachidonate (20:4 n-6), gondoate (20:1 n-9), docosanoate (22:0), nervonate (24:1 n-9), and total fatty acids. Metabolite concentrations were expressed as ng (bile acids and sterols) and μg (fatty acids) per mg of feces.
