# Supplementary material for: Impacts of Vincristine and Prednisolone Chemotherapy on the Canine Gut Microbiota in Dogs Undergoing Treatment for Lymphoma
Source: Vet Comp Oncol. 2025 May 6;23(3):388–400. doi: 10.1111/vco.13063 (PMC12353578; doi:10.1111/vco.13063)
Supplement: Supplementary file 2 — Data S2. R code used to process 16S rRNA amplicon sequencing data. [file VCO-23-388-s003.docx]

#8.18.2024-Canine_CHOP_Microbiome- Pre versus Post Chemotherapy Analysis

# Open the following packages:

library (devtools)

library(dada2)

library(here)

library(stringr)

library(readr)

library(tibble)

library(tictoc)

library(csv)

##################################

## RETRIEVE SEQUENCE FILE PATHS ##

fwdFPs <- list.files(path = "FILE_PATH", pattern = "_1.fastq", full.names = TRUE)

revFPs <- list.files(path = "FILE_PATH", pattern = "_2.fastq", full.names = TRUE)

# Extract Sample IDs from the file names by splitting on "_"

sample_ids <- str_split(basename(fwdFPs), "_", n = 1)

#####################################

## SEQUENCE TRIMMING AND FILTERING ##

plotQualityProfile(fwdFPs[1:4])

plotQualityProfile(revFPs[1:4])

# Now we will work on filtering data (removing low quality reads before we use these sequences to create ASV's).

filtFs <- here("filtered", paste0(sample_ids, "_F_filt.fastq"))

filtRs <- here("filtered", paste0(sample_ids, "_R_filt.fastq"))

# These next two commands tell you to name each file in the FiltF or FiltR object by the sample id's we pulled into a string in our earlier command.

names(filtFs) <- sample_ids

names(filtRs) <- sample_ids

tic()

out <- filterAndTrim(fwdFPs, filtFs, revFPs, filtRs, truncLen = c(240,160),

compress = TRUE, multithread = TRUE)

toc()

filt_trim_res <- tibble(SampleIDs = sample_ids, Input = out[,1], Filtered = out[,2])

filt_trim_res

#######################

## LEARN ERROR RATES ##

# Forward Reads

tic()

errF <- learnErrors(filtFs, multithread=TRUE)

toc()

# Reverse Reads

tic()

errR <- learnErrors(filtRs, multithread=TRUE)

toc()

# Review Error Plots

plotErrors(errF, nominalQ=TRUE)

#########################################

## Sequence Correction (ASV Inference) ##

tic()

dadaFs <- dada(filtFs, err=errF, pool = "pseudo", multithread=TRUE)

toc()

# reverse reads

tic()

dadaRs <- dada(filtRs, err=errR, pool = "pseudo", multithread=TRUE)

toc()

# inspect 'dada' objects

dadaFs[[1]]

dadaRs[[1]]

#######################

# Merge paired ends for ASVs

mergers <- mergePairs(dadaFs, filtFs, dadaRs, filtRs, verbose=TRUE)

# Inspect the merger data.frame:

View(head(mergers[[1]]))

###########################

## CREATE SEQUENCE TABLE ##

seqtab <- makeSequenceTable(mergers)

dim(seqtab)

# Inspect distribution of sequence lengths- this is a good QC step to make sure you don't have many very long or very short sequences- we shouldn't because we filtered earlier.

table(nchar(getSequences(seqtab)))

# Remove ASVs significantly shorter/longer than expected (quality control):

seqtab <- seqtab[,nchar(colnames(seqtab)) %in% 250:256]

#####################

## REMOVE CHIMERAS ##

seqtab.nochim <- removeBimeraDenovo(seqtab, method="consensus", multithread=TRUE, verbose=TRUE)

dim(seqtab.nochim)

sum(seqtab.nochim)/sum(seqtab)

# Export a copy of your sequence count table as a csv file:

write.csv(seqtab.nochim, file = "ASV_sequence_counts_per_sample.csv")

############################################

## OVERVIEW OF READS THROUGH THE PIPELINE ##

getN <- function(x) sum(getUniques(x))

track <- tibble(filt_trim_res, DenoisedFwd = sapply(dadaFs, getN), DenoisedRev = sapply(dadaRs, getN),

Merged = sapply(mergers, getN), NonChimeric = rowSums(seqtab.nochim))

head(track)

write_tsv(track, here("dada2_pipeline_reads.tsv"))

# Open needed packages for downstream analysis and visualization:

library (phyloseq)

library (vegan)

library (pairwiseAdonis)

library (taxa)

library (tidyr)

library (dplyr)

library (stringr)

library (ggplot2)

library (DESeq2)

library(tidyverse)

library(ggrepel)

library (compositions)

library (readr)

library (coin)

library(testthat)

library (dataprep)

library(remotes)

library (ggthemes)

library (ggpubr)

# Assigning taxonomy and creating your taxonomy table:

# We used the silva taxonomic classifier.

taxa <- assignTaxonomy(seqtab.nochim, "silva_nr99_v138.1_train_set.fa", multithread=TRUE)

taxa <- addSpecies(taxa, "silva_species_assignment_v138.1.fa")

# Check how your classifier worked to assign taxonomy to your ASVs in each sample you have sequencing for:

taxa.print <- taxa # Removing sequence rownames for display only

rownames(taxa.print)

head(taxa.print)

# Format your metadata table for use in phyloseq:

metadata_pre_post <- as.csv("METADATA_FILE_PATH", row.names = 1, header = TRUE, sep = ",", check.names = TRUE, stringsAsFactors = TRUE)

head(metadata_pre_post)

# Create phyloseq object:

ps_pre_post <- phyloseq(otu_table(seqtab.nochim, taxa_are_rows=FALSE),

sample_data(metadata_pre_post),

tax_table(taxa))

# Alpha Diversity:

# Calculating alpha diversity values:

rich_pre_post = estimate_richness(ps_pre_post, measures = c("Shannon", "Observed", "invsimpson"))

# Exported CSV file with alpha diversity values:

write.csv(rich_pre_post, file = "alpha_diversity_values_pre_post_chemo.csv")

# Beta Diversity Analysis

# Beta Diversity- Bray-Curtis (NMDS):

# NMDS Bray-Pre/Post-Color by TimePoint (pre vs post):

GP_pre_post = ps_pre_post

GP.ord_pre_post <- ordinate(GP_pre_post, "NMDS", "bray")

p1_pre_post = plot_ordination(GP_pre_post GP.ord_pre_post, color="TimePoint")

p1_pre_post = p1_pre_post + scale_color_manual(values=c("#a6cee3", "#1f78b4")) + theme_bw() + geom_point(size=3)

p1_pre_post

# Perform PERMANOVA for pre versus post chemotherapy samples:

pre_post.matrix<-as.matrix(seqtab.nochim[,2:764])##response variables in a sample x species matrix

# Note numbers in seqtab.nochim object will vary based on your individual table dimensions.

metadata_pre_post$TimePoint<-as.factor(metadata_pre_post$TimePoint)

pre_post.dist<-vegdist(pre_post.matrix, method='bray', na.rm = TRUE)

# Overall PERMANOVA using adonis2:

pre_post.div<-adonis2(pre_post.dist~TimePoint, data=metadata_pre_post, permutations = 999, method="bray")

# View the results of your overall PERMANOVA:

pre_post.div

#Export PERMANOVA results

write.csv(pre_post.div, file = "PERMANOVA_bray_curtis_pre_post_chemo.csv")

# Calculate Phylum percent abundances for each sample:

phylum_abundance_pre_post <- ps_pre_post %>%

tax_glom(taxrank = "Phylum") %>% # agglomerate at phylum level

transform_sample_counts(function(x) {(x/sum(x))*100}) %>% # Transform to rel. abundance

psmelt() %>% # Melt to long format

arrange(Phylum)

head(phylum_abundance_pre_post)

filtered_phylum_abundance_pre_post<-filter(phylum_abundance_pre_post, Abundance>=1) # removes ASVs with lower than 1% abundance

write_csv(phylum_abundance_pre_post, file = "Percent_abd_phylum_pre_post_chemo.csv")

write_csv(filtered_phylum_abundance_pre_post, file = "FILTERED_percent_abd_phylum_pre_post_chemo.csv")

# Calculate Family percent abundances for each sample:

family_abundance_pre_post <- ps_pre_post %>%

tax_glom(taxrank = "Family") %>% # agglomerate at Family level

transform_sample_counts(function(x) {(x/sum(x))*100}) %>% # Transform to rel. abundance

psmelt() %>% # Melt to long format

arrange(Family)

head(family_abundance_pre_post)

filtered_family_abundance_pre_post<-filter(family_abundance_pre_post, Abundance>=1) # removes ASV's with lower than 1% abundance

write_csv(family_abundance_pre_post, file = "Percent_abd_family_pre_post_chemo.csv")

write_csv(filtered_family_abundance_pre_post, file = "FILTERED_percent_abd_family_pre_post_chemo.csv")

# Differential Abundance Analysis with DeSeq2

##Deseq2- Comparison of Pre versus Post Chemotherapy Samples (Phylum Level):

ps.taxa_Phylum_pre_post <- tax_glom(ps_pre_post, taxrank = 'Phylum', NArm = FALSE)

Phylum_pre_post = phyloseq_to_deseq2(ps.taxa_Phylum_pre_post, ~ TimePoint_2)

Phylum_pre_post = DESeq(Phylum_pre_post, test="Wald", fitType="parametric")

res_Phylum_pre_post = results(Phylum_pre_post, cooksCutoff = FALSE)

res_Phylum_pre_post <- results(Phylum_pre_post, contrast=c("TimePoint_2", "post-chemo", "pre-chemo"))

head(res_Phylum_pre_post)

sigtab_Phylum_pre_post = res_Phylum_pre_post[which(res_Phylum_pre_post$padj <= 1.01), ]

sigtab_Phylum_pre_post = cbind(as(sigtab_Phylum_pre_post, "data.frame"), as(tax_table(ps.taxa_Phylum_pre_post)[rownames(sigtab_Phylum_pre_post) ], "matrix"))

head(sigtab_Phylum_pre_post)

write.csv(sigtab_Phylum_pre_post, file = "Sigtab_Phylum_pre_post_differential_abundance.csv")

##Deseq2- Comparison of Pre versus Post Chemotherapy Samples (Family Level):

ps.taxa_Family_pre_post <- tax_glom(ps_pre_post, taxrank = 'Family', NArm = FALSE)

Family_pre_post = phyloseq_to_deseq2(ps.taxa_Family_pre_post, ~ TimePoint_2)

Family_pre_post = DESeq(Family_pre_post, test="Wald", fitType="parametric")

res_Family_pre_post = results(Family_pre_post, cooksCutoff = FALSE)

res_Family_pre_post <- results(Family_pre_post, contrast=c("TimePoint_2", "post-chemo", "pre-chemo"))

head(res_Family_pre_post)

sigtab_Family_pre_post = res_Family_pre_post[which(res_Family_pre_post$padj <= 1.01), ]

sigtab_Family_pre_post = cbind(as(sigtab_Family_pre_post, "data.frame"), as(tax_table(ps.taxa_Family_pre_post)[rownames(sigtab_Family_pre_post) ], "matrix"))

head(sigtab_Family_pre_post)

write.csv(sigtab_Family_pre_post, file = "Sigtab_Family_pre_post_differential_abundance.csv")
