# Supplementary material for: Impacts of Vincristine and Prednisolone Chemotherapy on the Canine Gut Microbiota in Dogs Undergoing Treatment for Lymphoma
Source: Vet Comp Oncol. 2025 May 6;23(3):388–400. doi: 10.1111/vco.13063 (PMC12353578; doi:10.1111/vco.13063)
Supplement: Supplementary file 3 — Data S3. R code used to process Spearman’s correlation analysis. [file VCO-23-388-s005.docx]

library("corrplot")

library("readr")

library("dplyr")

library("tidyverse")

library("ggtext")

library("ggplot2")

library("reshape2")

library("viridis")

library("pheatmap")

library("psych")

library("ggcorrplot")

library("ggthemes")

#Pre-Chemo- DI and metabolite correlations:

# First, input data frame (make sure only numeric columns with variable headers):

pre_chemo_all_DI_metabolites <- read.csv("DATAFRAME_FILEPATH.csv")

#Convert dataframe into a matrix object:

pre_chemo_all_DI_metabolites <- as.matrix.data.frame(pre_chemo_all_DI_metabolites)

#Calculate r and p-values for all pairs of data in a matrix (can do as spearman, pearson, or kendall):

pre_chemo_all_DI_metabolites_spearman_p_values <- rcorr(pre_chemo_all_DI_metabolites, type = c("spearman"))

#Export p-values for Spearmans correlations:

write.csv(pre_chemo_all_DI_metabolites_spearman_p_values$P,"Pre_chemo_all_DI_metabolites_spearman_p_values.csv")

#Export r-values for Spearmans correlations

write.csv(pre_chemo_all_DI_metabolites_spearman_p_values$r,"Pre_chemo_all_DI_metabolites_spearman_r_values.csv")

#Post-Chemo- DI and metabolite correlations:

# First, input data frame (make sure only numeric columns with variable headers):

post_chemo_all_DI_metabolites <- read.csv("DATAFRAME_FILEPATH.csv")

#Convert dataframe into a matrix object:

post_chemo_all_DI_metabolites <- as.matrix.data.frame(post_chemo_all_DI_metabolites)

#Calculate r and p-values for all pairs of data in a matrix (can do as spearman, pearson, or kendall):

post_chemo_all_DI_metabolites_spearman_p_values <- rcorr(post_chemo_all_DI_metabolites, type = c("spearman"))

#Export p-values for Spearmans correlations:

write.csv(post_chemo_all_DI_metabolites_spearman_p_values$P,"Post_chemo_all_DI_metabolites_spearman_p_values.csv")

#Export r-values for Spearmans correlations:

write.csv(post_chemo_all_DI_metabolites_spearman_p_values$r,"Post_chemo_all_DI_metabolites_spearman_r_values.csv")
